# Enhanced antibacterial potential of exopolysaccharide-stabilized spice oil emulsions against foodborne pathogens

**DOI:** 10.3389/fnut.2025.1624274

**Published:** 2025-07-15

**Authors:** Jahnavi Kumari Singh, Steve Djiazet, Palanisamy Bruntha Devi, Horliane Nzali Ghomdim, G. Bhanuprakash Reddy, Digambar Kavitake, Prathapkumar Halady Shetty

**Affiliations:** ^1^Department of Food Science and Technology, Pondicherry University, Pondicherry, India; ^2^Université de Lorraine, CNRS, LRGP, Nancy, France; ^3^Bioprocess Laboratory, Department of Food Process and Quality Control, University Institute of Technology (IUT), University of Ngaoundere, Ngaoundere, Adamaoua, Cameroon; ^4^Division of Biochemistry, ICMR - National Institute of Nutrition, Hyderabad, India

**Keywords:** glucan exopolysaccharide, spice oil, emulsions, antimicrobial activity, food preservation

## Abstract

Exopolysaccharides (EPSs) from lactic acid bacteria (LAB) are known to have diverse applications in food and pharmaceuticals due to their functional properties. Emulsification is one of the important potentials of microbial polysaccharides, revealing its role as a natural emulsifier. In this study, glucan has been explored for emulsification with spice oils from *Xylopia aethiopica* (Xae), *Monodora myristica* (Mm), and *Fagara lepreuri* (Fl) Cameroon underutilized spices. Emulsifying ability, turbidity, emulsion droplet size, and micrographs of glucan-based spice oil emulsion were studied. The effect of sonication has been studied on droplet size and morphology of the emulsions. Sonication treatment has improved the emulsion stability by converting the larger emulsion droplets into smaller ones. The results of the disk diffusion assay confirmed antibacterial activity of emulsions, exhibiting high antibacterial efficiencies against food pathogens *Listeria monocytogenes* 1143, *Shewanella putrefaciens* 8104, and *Salmonella enterica* 950. This study underscores the potential of EPS as a natural emulsifying agent in food preservation, pharmaceuticals, and antimicrobial applications.

## Graphical Abstract

Emulsification of glucan exopolysaccharide with the Cameroonian spice oils and their anti-microbial potentials. 
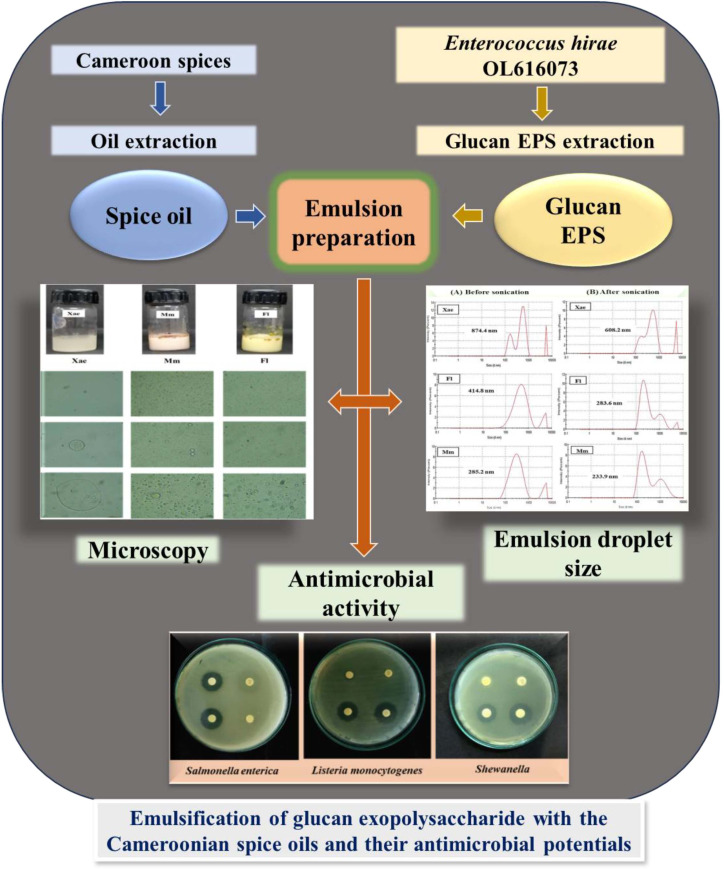

## Highlights

• Spice oils were emulsified with glucan exopolysaccharides extracted from *Enterococcus hirae* using the ultrasonication method.
• Ultrasonication enhanced emulsion turbidity, improved dispersion, homogeneity, and emulsion stability over 20 days.
• Nanoemulsions demonstrated significant antibacterial activity, particularly against foodborne pathogens.

## 1 Introduction

Natural preservatives derived from plants, animals, or minerals help extend the shelf life of food and other products by inhibiting microbial growth. Common examples include salt, vinegar, honey, and citrus extracts, which have been used for centuries to prevent spoilage. Unlike synthetic preservatives, such alternatives are often considered safer and less likely to cause adverse health effects. With growing consumer demand for clean-label and chemical-free products, natural preservatives, including spice oils, are progressively being used in the food, cosmetic, and pharmaceutical industries (1, 2). Spice oils are essential oils that are extracted from traditional aromatic plants. Spice oils, such as those from cloves, cinnamon, oregano, and rosemary, also serve as effective natural preservatives due to their strong antimicrobial and antioxidant properties. These oils are a homogenous mixture of organic chemical compounds from the same chemical family (3, 4).

Recent studies have proved the microbial inhibitory effects of such spices (5, 6). Cameroonian spice oils have been known to possess antibacterial, antioxidant, anti-plasmodial, and insecticidal activities (7, 8). Three chosen spices, *Xylopia aethiopica (Xae), Monodora myristica (Mm), and Fagara leprieurii (Fl)*, are traditionally valued in Cameroon, not just for their flavor but also for their preservation and medicinal properties. Their cultural and functional context underlines their importance as natural preservatives (9). The oil contents of these spices are 34% for *X. aethiopica*, 53% for *M. myristica*, and 33% for *F. leuprieurii* (10). Incorporation of such spice oils as a preservative is limited by its strong odor and hydrophobicity, which can be overcome by the use of nanotechnology (11, 12). Plant-derived essential oils can be converted to water water-dispersible form by encapsulating them using oil, water, and a proper emulsifier (13). Formulations used in studies of soy-lecithin or polysorbate-stabilized nanoemulsions with other plant-derived oils and natural emulsifiers (e.g., lecithin, gum arabic, saponins) have been shown to enhance stability and antimicrobial delivery compared to systems using synthetic surfactants like Tween 20. The smaller the size of the emulsion droplets, the more the advantage, as it can give better physical stability, good appearance, release kinetics, resistance to degradation and increased biological activity than conventional emulsions (14–16).

Nanoemulsions being the smaller size of emulsions prepared up-to-date, are a class of emulsions with droplet sizes from 20 to 100 nm (6, 17). Because of the small droplet size, nanoemulsions appear transparent or translucent and are more stable with respect to creaming, coalescence, flocculation, and Ostwald ripening than conventional emulsions (18). The physicochemical properties of nanoemulsions are interesting for practical applications because of the small droplet size and long-term stability (19–21).

Nanoemulsion can be prepared by using different high energy and low energy methods including ultrasonication (22, 23). The formation of nanoemulsion is controlled by the relationship between droplet disruption and droplet coalescence (24, 25). The ultrasonic processor applies excellent shear force for droplet disruption, and the rate of droplet coalescence is determined by the mixed exopolysaccharide (EPS) and its concentration (26, 27). There are two main mechanisms operating during ultrasonic emulsification (28). First, an acoustic field produces interfacial waves to break the dispersed phase into the continuous phase (29). Second, the formation of acoustic cavitation is used to collapse micro-bubbles into droplets of nanometric size by pressure fluctuations (30, 31). For proper emulsification, a good emulsifier should be used which shall provide high stability, resulting in good emulsification (15). The given bacterial EPS-producing strain was isolated from an acidic fermented food, further identified as *Enterococcus hirae* OL616073. The EPS has been extracted as and characterized as glucan containing α-(1→6) and α-(1→3) glycosidic linkage with a backbone of glucose subunits (32). Glucan EPS was chosen over other polysaccharides because the porous, spongy, granular morphology that was observed under scanning electron microscopy, also having strong water solubility index (76.75%), water water holding capacity (296.19%), oil holding capacity (379.91%), and emulsifying activity (EA1- 72.22%). High yield (20 g/L) and physico-functional properties of this EPS have an advantage over other microbial polysaccharides (33). So, to achieve the demand for natural preservation, spice oils and bacterial EPS are used as a preservative and their various biological activities (anti-bacterial, anti-biofilm and minimum inhibitory concentration) are checked against clinically important food pathogens (34, 35).

## 2 Materials and methods

Three Cameroonian under-utilized spices *X. aethiopica* (Xae), *M. myristica* (Mm), and *F. lepreuri* (Fl) were purchased from Bamenda and Bafoussam markets in Cameroon. Glucan EPS was produced and extracted as reported earlier and used in this study (32). The chemicals, media, antibiotic disks, and enzymes used in this work were of analytical grade and purchased from Hi-Media (Mumbai, India) and Sigma-Aldrich Merck (United States). The reference pathogenic strains causing foodborne illness including *Listeria monocytogenes* MTCC 1143, *Shewanella putrefaciens* MTCC 8104 and *Salmonella enterica* 950 were procured from the Microbial Type Culture Collection (MTCC) and Gene Bank, Institute of Microbial Technology, Chandigarh, India.

### 2.1 Spice oil extraction

The spices samples were ground using an electric blender (BTC Cast Iron Domestic SS Cup Hand Grinder). About thirty grams of each sample was folded in filter papers and put in extraction timbers. The oil extraction was done using Soxhlet, at 65°C for four hours. The extraction solvent was hexane. After extraction, the oil was dried 45°C to evaporate hexane. It was then stored for further analysis.

### 2.2 Glucan EPS production

*Enterococcus hirae* OL616073 was isolated from 14 h fermented idli batter and used for the production of glucan EPS. The EPS extraction was carried out as per the method explained in earlier reports and used to develop the spice oil emulsions in this study (32, 36).

### 2.3 Emulsion preparation

A set of preliminary trial experiments were performed to demonstrate the emulsifying activity and efficacy prior to the main study. Spice oil emulsions were prepared in a ratio of 3:1 (EPS: Spice oil), where 1% glucan EPS solution with distilled water and three spice oils (Xae, Mm, and Fl) were involved separately. In first phase, the suspension was stirred homogenously and vortexed for 30 min at 40 Hz (Top mix FB 15024, Fisher Scientific, United Kingdom) to make the coarse emulsion. And in second phase, coarse emulsion was subjected for 10 min sonication (model: Labman Ultrasonicator). Coarse and ultrasonicated emulsions were further analyzed in this study.

### 2.4 Turbidity of emulsions

Exopolysaccharide based spice oil emulsions were diluted 40 times with phosphate buffer solution and phosphate buffer was used as the blank. Absorbance was measured using an LW-1600FC UV spectrophotometer (Shanghai Jinghua Technology Equipment Company, Ltd., Shanghai, China) at 600 nm. Turbidity (T) was calculated as in Li et al. (37) method, using following formula:


T=2.302×(A×V)/I

A = Absorbance of diluted emulsion at 600 nm measured against the blank. V = Dilution (40).

I = Optical path length (1 cm).

### 2.5 Microscopy

Before and after sonication, all emulsions were examined at 10, 20 and 40× magnification lenses under Inverted Routine Microscope (Nikon eclipse TS100 microscope) using cavity microscope slides (38).

### 2.6 Particle size analysis

Spice oil emulsions were subjected for average particle size distribution measurement by using a nanoparticle size analyzer (Zetasizer, United Kingdom) at 25°C.

### 2.7 Antimicrobial potential of emulsions

Antimicrobial assay was performed against Gram-positive (*Listeria monocytogenes* MTCC 1143) and Gram-negative (*Shewanella putrefaciens* MTCC 8104 and *Salmonella enterica* MTCC 950) pathogenic strains. Using aseptic procedures, overnight incubated broth cultures of *Listeria monocytogenes*, *Shewanella putrefaciens* and *Salmonella enterica*, were spread to form microbial growth lawns on Mueller Hinton agar plates (Himedia, India). The sterile disks were evenly spaced on the surface of the inoculated agar plates and impregnated drop by drop with 20 μl of emulsions. Standard antibiotic disks of Cephoxitin (CX30) were considered as positive control for the assay. Disks were allowed to dry for 15 min to initiate uniform diffusion of emulsion into the agar and then inverted and incubated for 24 h at 37°C. The next day zone of inhibition were recorded in mm around the disks in each plate (16). Appropriate spice oil controls for all three emulsions against the reference pathogenic strains were considered in well diffusion assay performed as per the aforementioned protocol.

### 2.8 Statistical analysis

All the experiments were carried out in triplicates and the results were represented as mean ± SD.

Graphs were plotted using Microsoft Office 2016, GraphPad Prism 8.0.2, and OriginPro 8 software.

## 3 Results and discussion

### 3.1 Turbidity measurement of emulsions

**FIGURE 1:**
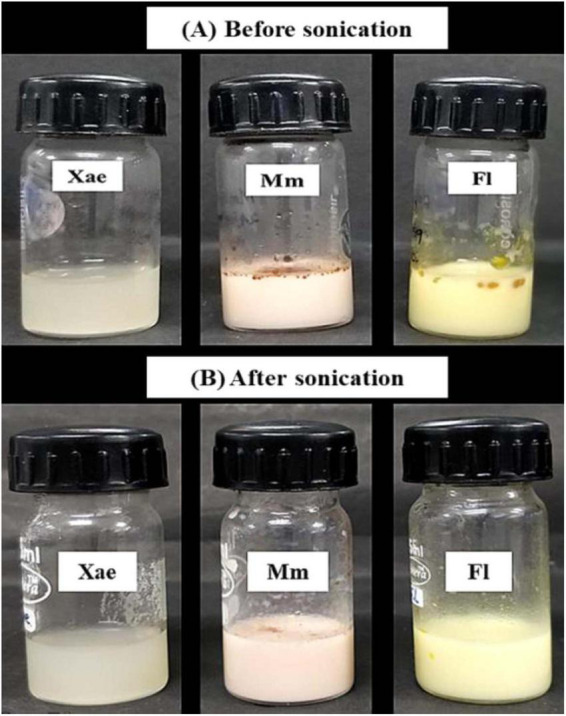
Photographs of spice oil emulsions, **(A)** before sonication and **(B)** after sonication.

Spice oil emulsions were prepared with glucan EPS in two phases. There was a clear difference in the clarity, turbidity and homogeneity of the emulsions before and after sonication (Figure 1). Emulsion turbidity is directly proportional to the emulsion stability and inversely proportional to the size of emulsion droplets (39).

The turbidity of the coarse and sonicated EPS-based spice oil emulsions is recorded in Table 1. It provides insights into the turbidity and stability of various spice oil emulsions across two phases, before and after sonication. Before sonication Xae, Fl, and Mm emulsions showed a turbidity of 902.384 ± 16.2, 2521.457 ± 28.1, and 2463.446 ± 24.8 which increased after the sonication treatment to 1043.573 ± 12.8, 2695.795 ± 24.3, and 3494.742 ± 32.4 respectively. A one-way Analysis of Variance (ANOVA) followed by Tukey’s Significant Difference (HSD) *post-hoc* test was performed to compare turbidity values among the three emulsions (Xae, Mm, and Fl) within each phase. Superscript letters (a–d) were added to the data table to indicate statistically distinct groups. This moderate increase suggests that the Xae emulsion becomes relatively more stable after sonication, likely due to the breakdown of the emulsion droplets leading to the smaller emulsion droplet size (40).

**TABLE 1:** Turbidity of spice oil emulsions.

Spice oil emulsion	Turbidity (mean ± SD)	
	Phase I	Phase II
Xae	902.384 ± 16.2^b^	1043.573 ± 12.8^c^
Mm	2463.446 ± 24.8^a^	3494.742 ± 32.4^a^
Fl	2521.457 ± 28.1^a^	2695.795 ± 24.3^b^

Different superscripts (a–c) are significantly different (*p* < 0.05) with each other, column-wise.

### 3.2 Microscopic observation

**FIGURE 2:**
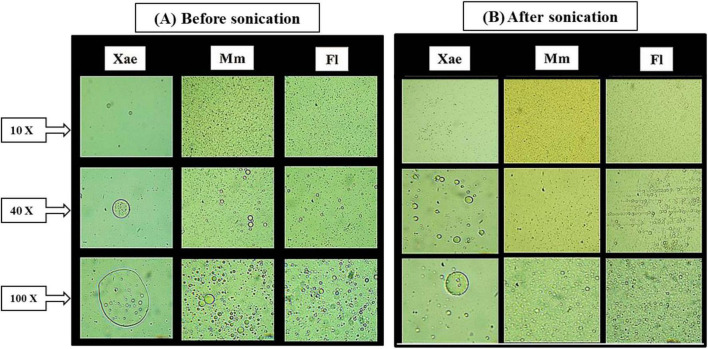
Micrographs of spice oil emulsions, **(A)** before sonication and **(B)** after sonication.

The morphology of spice oil emulsion droplets was observed before and after the ultrasonication process under an Inverted Routine microscope. Figure 2 reveals that emulsions subjected to ultrasonication exhibit significantly smaller droplet sizes than their pre-sonication counterparts. This reduction in droplet size is evident across all the spice oil emulsions (Xae, Mm, and Fl), indicating the effectiveness of ultrasonication assisted break down of larger droplets into finer and uniform structures. The reduced droplet size and improved homogeneity post-sonication, as seen under magnification lenses are consistent with previous studies which have demonstrated its effectiveness in reducing droplet size increasing the emulsion stability by enhanced dispersion of the oil phase within the aqueous phase (41, 42). The phenomenon can be explained by the cavitation effect, where ultrasonic waves create microscopic vapor cavities in the liquid medium which collapse eventually generating an intense localized energy that disrupts larger droplets into smaller ones (43). The mechanical shear forces generated by ultrasonic waves are known to form uniform structures, thereby enhancing the stability of the emulsion (23).

### 3.3 Particle size analysis of emulsion

**FIGURE 3:**
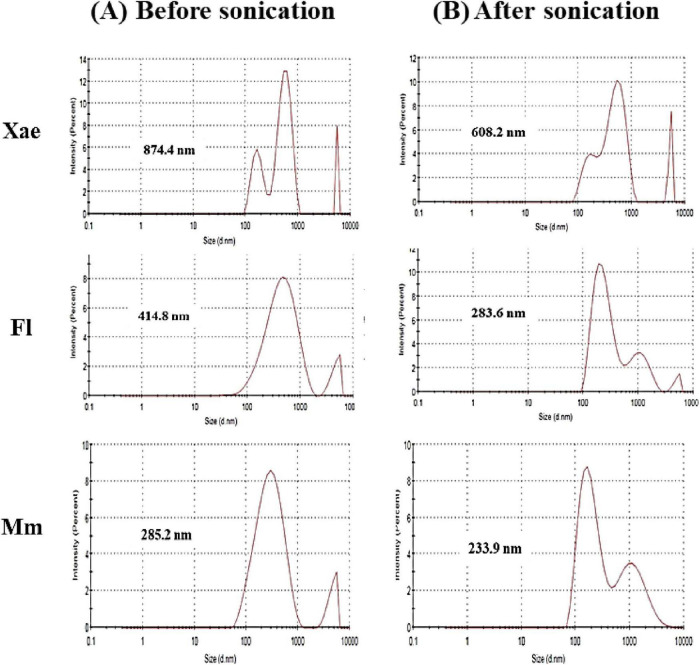
Particle size distribution of spice oil emulsions, **(A)** before sonication and **(B)** after sonication.

Colloidal stability, turbidity and other properties of food systems are highly influenced by the size distribution emulsion droplets. Figure 3 highlights the particle size distribution of spice oil nanoemulsions (Xae, Fl, and Mm) before and after sonication, underscoring the transformative impact of sonication on emulsion properties. For the Xae nanoemulsion, the particle size reduced from 874.4 nm before sonication to 608.2 nm after sonication, showcasing a significant improvement in size uniformity. Similarly, the Fl nanoemulsion reduced from 414.8 to 283.6 nm, while the Mm nanoemulsion decreased from 285.2 to 233.9 nm. PDI (Polysidpersivity index) was also revealed in this study. Before sonication, Xae, Fl, and Mm emulsion had PDI of 1, 0.393, and 0.391 which was changed after sonication to 0.669, 0.382, and 0.412 respectively. This consistent reduction in particle size across all emulsions suggest disruption of larger oil droplets into smaller and more stable particles after sonication. The observed trends align with the known effects of sonication, which involves high-energy ultrasonic waves that generate intense shear forces, breaking down oil droplets into nanoscale dimensions. The Mm nanoemulsion showed the smallest particle size both before and after sonication, indicating its inherent stability, potentially due to its composition or initial processing conditions. Sonication also likely contributes to better stabilization by improving the interaction between the oil phase and emulsifying agents, reducing the potential for droplet coalescence and phase separation. The enhanced emulsification, particularly in the Mm nanoemulsion, suggests its suitability for applications where fine particle sizes are critical, such as in targeted drug delivery systems or high-performance food formulations. Smaller particle sizes lead to better surface area-to-volume ratios, which enhance the emulsifying capacity and stability of nanoemulsions, as previously observed in studies (44). These results highlight the effectiveness of sonication in optimizing nanoemulsion characteristics and its potential applications in food, pharmaceutical, and cosmetic based industries which require stable and uniform emulsions (14, 19).

### 3.4 Antimicrobial activity of EPS based nanoemulsions

**FIGURE 4:**
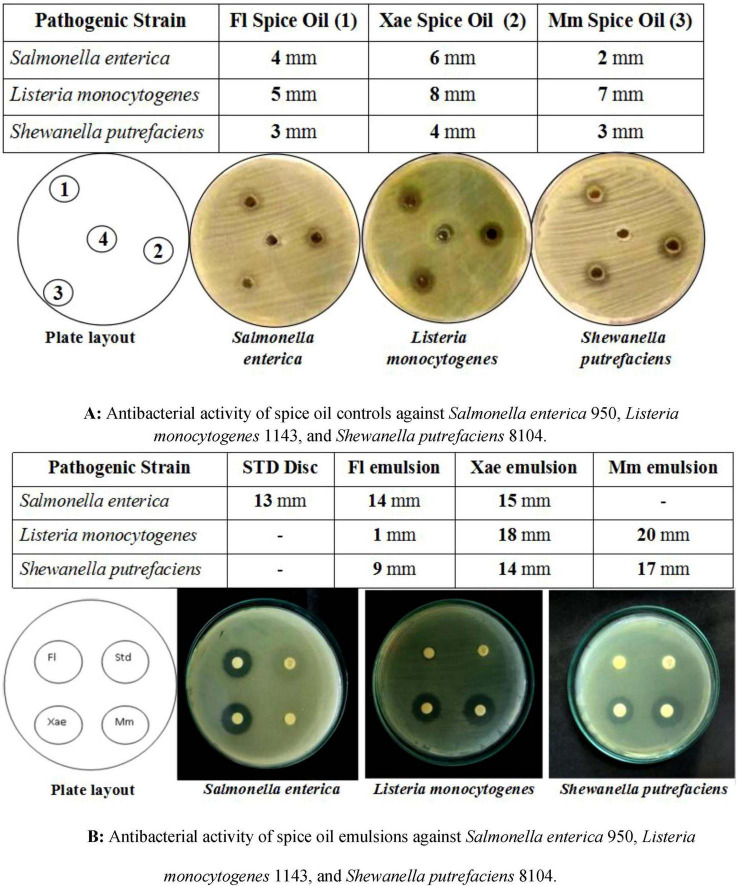
**(A)** Antibacterial activity of spice oil controls against *Salmonella enterica* 950, *Listeria monocytogenes* 1143, and *Shewanella putrefaciens* 8104, **(B)** antibacterial activity of spice oil emulsions against *Salmonella enterica* 950, *Listeria monocytogenes* 1143, and *Shewanella putrefaciens* 8104.

The antibacterial activity of spice oil alone (Figure 4A) and spice oil emulsions (Figure 4B) against *Salmonella enterica, Listeria monocytogenes*, and *Shewanella putrefaciens* reveals a significant enhancement when emulsified with EPS. The primary observations from both figures highlight the increased inhibition zone diameters with emulsified spice oils compared to their free oil counterparts, signifying the improved antibacterial efficacy. Fl emulsion showed an average inhibition of 9 mm in *Shewanella putrefaciens*, 14 mm inhibition for *Salmonella enterica* and 1 mm inhibition for *Listeria monocytogenes*. Emulsion of Mm showed inhibition for 17 mm in *Shewanella putrefaciens* whereas resulted in an enhanced inhibition of 20 mm in *Listeria monocytogenes*. It did not show any inhibition for *Salmonella enterica* which may be due to the less antimicrobial activity or low level of diffusion in the agar matrix. Xae emulsion showed clear zone of inhibition of diameter 14 mm against gram negative *Shewanella putrefaciens*, 15 mm for *Salmonella enterica*, and maximum inhibition of 18 mm for *Listeria monocytogenes*. The spice oil emulsions demonstrated significant antibacterial activity that is in agreement with earlier findings (45). The enhanced activity of emulsified spice oils likely results from converging mechanisms like increased surface area diffusion, encapsulation and sustained release, synergistic interface interaction and biofilm interferences. In a previous study, similar oil emulsions and nanoemulsions containing phenolic compounds like thymol and eugenol have shown antimicrobial effects on food pathogens like *Listeria monocytogenes*, *Escherichia coli*, and *Bacillus subtilis* by inducing cell lysis and stimulating bacterial envelope damage (46).

The inhibition zones of spice oils alone (Figure 4A) ranged between 2 and 8 mm, indicating moderate antibacterial activity. In contrast, EPS-based emulsions (Figure 4B) significantly enhanced antibacterial action, with inhibition zones ranging from 9 to 20 mm. For *Listeria monocytogenes*, the individual spice oils showed relatively weak inhibition (≤ 8 mm), while the emulsions of Xae and Mm spice oils exhibited much higher inhibition zones (18 and 20 mm, respectively). This suggests that emulsification improved the bioavailability and antimicrobial effectiveness against this strain. The spice oil alone showed very weak activity against *Shewanella putrefaciens*, with inhibition zones not exceeding 4 mm. However, the emulsions resulted in significantly larger inhibition zones, especially with Mm (17 mm) and Xae (14 mm) emulsions, demonstrating that emulsification enhances antimicrobial action even against more resistant strains. The STD disk for *Salmonella enterica* showed an inhibition zone of 13 mm, while Fl and Xae emulsions exceeded this (14 and 15 mm, respectively). This suggests that EPS-based emulsions can potentially match or even outperform conventional antibacterial agents. Essential oils and spice oils are hydrophobic in nature, limiting their solubility and diffusion in aqueous environments. EPS-based emulsions improve the dispersion of spice oils, leading to better contact with bacterial cells and increased antimicrobial efficacy (47, 48). Emulsification helps in the controlled release of antimicrobial compounds, leading to prolonged activity and reducing the need for high concentrations of spice oil, which can sometimes affect sensory properties in food applications (49, 50).

## 4 Conclusion and future perspectives

This study successfully developed stable nanoemulsions incorporating glucan EPS and spice oils from Cameroonian spices using ultrasonication. The nanoemulsions exhibited enhanced stability, reduced droplet size, and promising antimicrobial activity against food pathogens. By addressing challenges such as the strong odor and hydrophobicity of spice oils, this approach highlights the utility of nanotechnology in enhancing the practical applicability of natural bioactive compounds in food safety and preservation systems. EPS-based emulsions of spice oils demonstrate superior antibacterial activity compared to spice oils alone. This enhanced efficacy is likely due to improved solubility, bioavailability, and controlled release of antimicrobial compounds. These findings align with earlier studies reporting the antimicrobial synergy of EPS and essential oils. With toxicological safety profiles, stability under physiological conditions, and *in vivo* efficacy studies these microbial EPS-based emulsions would be potent drug carriers (e.g., targeted or sustained delivery). This underscore the potential of glucan-based EPS as a natural, multifunctional emulsifier with both stabilizing and antimicrobial properties, supporting its application in food preservation, pharmaceutical delivery, and functional formulations.

## Data Availability

The original contributions presented in this study are included in this article/supplementary material, further inquiries can be directed to the corresponding authors.
